# Prevalence and risk factors for under nutrition among children under five at Haramaya district, Eastern Ethiopia

**DOI:** 10.1186/s12887-015-0535-0

**Published:** 2015-12-16

**Authors:** Hiwot Yisak, Tesfaye Gobena, Firehiwot Mesfin

**Affiliations:** Debretabor University, College of Health and Medical Science, P.O. Box 272, Debre Tabor, Ethiopia; Harmaya University, College of Health and Medical Science, P.O. Box 235, Harar, Ethiopia

**Keywords:** Stunting, Wasting, Underweight, Haramaya District

## Abstract

**Background:**

Under nutrition is one of the major causes of health problems among children under five years old in Ethiopia. Though the problem of under nutrition has decreased in the country, it is still continuing as one of the major causes of mortality of children under five. Studies have shown that the magnitude and related factors of under nutrition are varied in different agro-ecological settings of the country. Thus it is indispensable to assess the nature of the problem at community level. The objective of this study was to assess the extent of under nutrition and related factors among children under five years in Haramaya district, eastern Ethiopia.

**Methods:**

A community based cross sectional study was conducted in Haramaya district from December 1, 2012 to January 30, 2013 and Multi–stage stratified systematic random sampling technique was used to select the study subjects. A total of 791 study subjects were included in the study. Data were collected using face-to-face interview and anthropometric measurements. World Health Organization (WHO) Anthro software was used to convert nutritional data indices from anthropometric measurement into Z-scores, and Multivariate logistic regression model with an enter method was used to determine the predictors of under nutrition.

**Results:**

The study indicated that prevalence of stunting, wasting and underweight among children under five years old were 45.8 %, 10.7 % and 21 % respectively. Children in rural Kebeles with Adjusted odd ratio (AOR) =2.45, 95 % CI(1.25-6.66), children who were 6 and above birth order (AOR =1.992, 95 % CI( 1.05-3.77)), and children who were used to live with households having two and more under five children (AOR = 1.81, 95 % CI( 1.19-2.7)) were more stunted than their counterparts. Children in the lowland Kebeles, (AOR = 3.29, 95 % CI( 1.2-8.8)) and children having diarrhea, (AOR = 2.48, 95 % CI(1.28-4.78)); mothers with Body mass index (BMI) < 18.5 (AOR = 2.17, 95 % CI(1.17-3.81)); mothers who did not have ANC visit during pregnancy (AOR = 3.47, 95 % CI (1.49-7.8) ) and with birth order of 4 to 5 children (AOR = 3.08, 95 % CI (1.11-8.5)), were more likely to be underweight than their counterparts. Moreover, male children (AOR = 2.37, 95 % CI (1.19-4.7)), children who were served food with family (AOR = 2.3, 95 % CI (1.14- 4.9)), children who had fever, (AOR = 2.9, 95 % CI (1.16-7.2)), were more likely to be wasted than their counterparts.

**Conclusions:**

This study indicated that nearly half of the children under five years in the study area were stunted. Thus, a large number of children had poor nutritional history or growth failure. Furthermore, underweight and wasting were significantly high. The problem can be addressed by targeting children since their early ages and by conducting tailored nutrition education to mothers or caretakers to improve the nutritional status of their children.

## Background

Malnutrition generally implies both a state of under nutrition and over nutrition [1].The consequences of under nutrition among under-five children are mortality and illness, intelligence loss and reduced productivity and also it is inter-generational [2]. In Ethiopia, under nutrition is one of the major health problems among children under five years of age. Though the problem of under nutrition has decreased in the country, it is still continuing as one of the major causes of mortality. However, studies in the country have shown that the magnitude and the underlying factors of under nutrition among under five children vary across different agro-ecological settings [2, 3].

Therefore, it is indispensable to assess the problem at community level using community based analytical cross sectional study to determine the underlying causes of the problem and to design appropriate strategies which can be helpful in reducing the problem. The objective of the study was to assess the prevalence of the problem and other factors related to under nutrition among children who are under five years in the target area.

## Methods

### Study area

The study was conducted in Haramaya district, eastern Ethiopia. Haramaya is one of the districts in the Oromia Regional State of Ethiopia. It is found in eastern Hararge zone and located about 506 km east of the capital, Addis Ababa. In the district, there are five urban and 33 rural Kebeles (small sub divisions of a district). Of these, 12 kebeles are located in lowland areas and the remaining 26 are in mid land. Its (the district’s) altitude ranges from 1400 to 2340 meters above sea level.

#### Study design and population

Community- based cross-sectional study was conducted in the district from December 1, 2012 to January 30, 2013. The study population was children under five years of age residing in the selected Kebeles of the district.

#### Sample size and sampling techniques

The sample size for the study was determined by epi info version 3.2 by considering the difference of proportion of stunting in rural (46 %) and in urban areas (32 %) (CSA, 2011), and a 95 % Confidence interval, and Power of 80 %, i.e. (1-B) =0.80, Z 1-B = 0.84; a total of 798 children under five years of age were included in the study. The sample size was large enough to determine both the extent of the under nutrition and its related factors among the study subjects.

The study employed multistage proportional stratified sampling technique. Of the total kebeles, two urban and four rural kebeles were randomly selected. Then, the study subjects were selected in proportion to the size of the study population of each kebele using systematic random sampling.

### Data collection

Data were collected by using semi-structured questionnaire adapted from Ethiopian National Nutrition Survey Questionnaire, and anthropometric measurements were also done. In households who had more than one children who were under five, one of them was selected randomly. Weights of the mother and the child were measured using united Nation’s International Children's Fund (UNICEF) SECA portable, digital scale with a capacity of 150kg to the nearest 0.1 kg., and measurement of height/length was done in a lying position with wooden board for children under two years of age and children above two years and mothers were measured in a standing position with centimeters to the nearest of 0.1cm. Twelve nurses who had diploma certificate and two other Nurses with BSc were involved in the data collection and supervision processes. The questionnaire was translated into local languages (Oromiffa and Amharic) for the field work and back to English to check its consistency. Both the interviewers and supervisors were trained for three days since they all have previous experience of anthropometric measurements and then pre-test was conducted in the neighboring Kebeles. Weighing scales were calibrated with known weight object regularly. Weight and height were measured twice by different persons and the mean value was used for the analysis. standardization test was conducted to see whether the data collectors have good precision and accuracy and fortunately the precision and the accuracy of most of the enumurators were acceptable. For those having poor accuracy retraining was given.

#### Data processing and analysis

The pre-coded data were entered into EpiData Version 3.2 and WHO Anthro software was used to convert nutritional data into Z-scores of the indices by using the new WHO growth standard. Children whose height-for-age, weight- for- height and weight- for- age < -2 SD from the median of the reference population were considered stunted, wasted and underweight respectively. Then, the data were exported to statistical package for social sciences (SPSS) software Version 16 for data processing and analysis. Crude odds ratio with 95 % confidence interval was used to assess the association between independent and dependent variables. Independent variables which had association with the outcome variable in the bivariate logistic regression and those with *P* value of <0.2 were considered candidate for the final logistic regression model. The Hosmer-Lemeshow test was used to check goodness of model fitting. Finally, an enter method was used to run the final multivariate a cut off point for statistical significance.

### Ethical considerations

Ethical clearance was obtained from Haramaya University, Harar Campus Institutional Research Ethics Review Committee. Prior to data collection, the interviewers had explained the objective, benefit and risks of the study to get informed written consent from mothers or care givers of the children. Data collectors gave advice to mothers or care givers of the under nutrition child to provide additional balanced diets to their children and to visit the nearby health facility.

## Results

A total of 791 under five children with their mothers/caregivers were involved in the study, which made the response rate 99.1 %. Majority of the respondents, were Oromo in their ethnicity (717(90.6 %)), and Muslims in their religion (695(87.9 %)). About half, (410(51.8 %)) of the surveyed households had two or more under five children. A total of 450(56.9 %) of the respondents and 391(49.4 %) fathers of the children were illiterate (Table 1).

**Table 1:** Socio demographic characteristics of study participants by place of residence in Haramaya woreda, Ethiopia, 2013

Socio demographic variables		Place of residence					
		Urban	%	Rural	%	Total	%
Head of	Male	207	87.3	425	76.7	632	79.9
House hold hold	Female	30	12.7	12.9	23.3	159	20.1
Ethnicity	Oromo	168	70.9	549	99.1	717	90.6
	Amhara	53	22.4	4	0.7	57	7.2
	Other	16	6.7	1	0.2	17	2.2
Religion	Muslim	144	60.8	551	99.5	695	87.9
	Orthodox	77	32.5	3	0.5	80	10.1
	Protestant	16	6.8	0	0	16	2
Family size	2-5	154	65	246	44.4	400	50.6
	6-12	79	33.3	305	55.1	384	48.5
	>12	4	1.7	3	0.5	7	0.9
Education	Illiterate	63	26.6	387	69.9	450	56.9
status the	Read & write	4	1.7	26	4.7	30	3.8
Mother	1-8	75	31.6	141	25.5	216	27.3
	9-12	42	17.7	0	0	42	5.3
	>12	53	22.4	0	0	53	6.7
Education	Illiterate	22	9.3	369	66.6	391	49.4
al status of	read& write	0	0	32	5.8	32	4
the father	1-8	52	21.9	145	26.2	197	24.9
	9-12	78	32.9	4	0.7	82	10.4
	>12	85	35.9	4	0.7	89	11.3
Occupatio	House wife	122	51.5	451	81.4	573	72.4
n of the	Farmer	5	2.1	68	12.3	73	9.2
Mother	Merchant	56	23.6	30	5.4	86	10.9
	Employed	54	22.8	5	0.9	59	7.5
Occupatio	Farmer	44	18.6	519	93.7	563	71.2
n of the	Government	79	33.3	7	1.3	86	10.9
father	Employee						
	Merchant	47	19.8	17	3.1	64	8.1
	Other employee	67	28.3	11	2	78	9.9

The mean age of surveyed children was 22.43 ± (1.27) months. Of the total, 449 (56.8 %) children were males and the rest were females, and almost all of the surveyed children, 773 (97.7 %) were single in their birth type and 753 (95.2 %) had taken vaccination. But, more than two third (572 (72.3 %)), of the children were born at home and the rest 219 (27.7 %) were born at health facilities. In the previous two weeks of the survey, a total of 111 (14 %) and 85 (10.7 %) of the under five children had diarrhea and fever respectively.

### Prevalence of under nutrition among under five children

The prevalence of wasting (WHZ score < -2) was 10.7 % in urban and 11.4 % in rural areas**.** About 45.8 % were stunted (HAZ score < -2), and of this, 30.8 % were urban residents, and 52.2 % were rural residents. The prevalence of underweight (WAZ Z score < -2) among children was 21 % ( 22 % in rural and 18.6 % in urban). The total rates of the prevalence of severe wasting (WHZ Score < -3), severe stunting (HAZ Z score < -3) and severe underweight (WAZ Score < -3) were 5.2 %, 31.6 %, and 5.6 % respectively (Table 2).

**Table 2:** Nutritional status of children and their mothers by pace or residence, in Haramaya District, Eastern Ethiopia, 2013

		Urban		Rural		Total	
		Frequency	percentage	frequency	percentage	frequency	percentage
Wasting status	Not wasted	215	90.7	491	88.6	706	89.3
	Wasted	22	9.3	63	11.4	85	10.7
	Total	237	100	554	100	791	100
Stunting status	Not stunted	164	69.2	265	47.8	429	54.2
	Stunted	73	30.8	289	52.2	362	45.8
	Total	237	100	554	100	791	100
Underweight status	Not underweight	193	81.4	432	78	625	79
	Underweight	44	18.6	122	22	166	21
	Total	237	100	554	100	791	100
Mothers Nutritional status	Overweight(BMI > 25)	35	15.2	18	3.2	53	68
	Normal (BMI 18.5-24.9)	177	76.6	437	78.9	614	78.2
	Underweight (BMI < 18.5)	19	8.2	99	17.9	118	15
	Total	231	100	554	100	785	100

### Factors associated with stunting

On bivariate analysis, children living in rural Kebeles were more likely to be stunted than those who live in urban areas and the crude odd ratio (COR) is 2.5, 95 % CI (1.7-3.3); Children living in lowland agro ecology were more likely to be more stunted than those living in highland (COR = 1.66, 95 % CI (1.3-2.2)); and illiterate mothers were more likely to have stunted child (COR = 3.55, 95 % CI (1.5-7.8)). Families earning less than 500 birr per month were more likely to have stunted child with COR = 2.5, 95 % CI (1.72-3.5), and lacking of farm land is associated with stunting (COR = 2.2, 95 % CI (1.56-3)). Male children were more likely to be stunted (COR = 1.6, 95 % CI (1.2-2)); high birth order children were more likely to be stunted (COR = 2.3, 95 % CI (1.4-3.7)). Children having diarrhea were more likely to be stunted with COR = 1.53, 95 % CI (1.02-2.3) than those who did not have. And underweight mothers were more likely to have stunted child with COR =3, 95 % CI (2-4.6) and using unprotected source of water was also associated with stunting (COR =2.6, 95 % CI (1.8-3.9)). While the confounders (educational status and occupational status) were controlled, stunting was higher among under five children in the rural kebeles with AOR = 2.45, 95 % CI (1.25-6.66). Children who were 6 and above birth order (AOR = 1.992, 95 % CI (1.05-3.77)), children who used to live in households who have two and more under five children (AOR = 1.81, 95 % CI (1.19-2.7)), children having mothers who were underweight BMI < 18.5 (AOR = 2.68, 95 % CI (1.68-4.27)), and children in the households using water from river (AOR = 1.95, 95 % CI (1.12-3.38))were more stunted than their counterparts (Table 3).

**Table 3:** Predictors of under nutrition among children of under five years of age in Haramaya district, eastern Ethiopia, 2013

Variables	Underweight		Wasting		Stunting	
	COR (95 % CI)	AOR(95 % CI)	COR (95 % CI)	AOR(95 % CICI)	COR (95 % CI)	AOR(95 % CI)
Place of residence						
Urban	1		1		1	
Rural	1.239(0.844-1.819		1.254(0.752-2.09)		2.45(1.78-3.38	2.88(1.25-6.6)**
Number of under fives						
1	1	1	1	1	1	1
> = 2	1.492(1.054-2.112	1.07(0.56-2.05)	2.29 (1.4-3.7	1.49(0.7-3.3)	1.07 (0.8-1.4)	1.8( 1.19-2.71)**
Age of the Child(in month)						
0-6	1	1	1		1	1
7-12	0.594(0.251-1.41)		0.3(.08- 1.101)		1.7 (1.1-2.97)	1.12(0.61-2.07)
13-24	0.749(0.406-1.379)		0.54(0.3-1.1		1.8 (1.14-3.1)	1.9(1.05-3.48)**
25-36	0.983(0.552-1.75)		0.8 (0.4-1.6)		2.08(1.2-3.5)	2.6(1.14-3.89)**
37-59	1.194(0.65-2.18)		0.5(.24-1.1)		1.9 (1.01-3.8)	1.74(0.81-3.7)
Birth order of the child						
1	1	1	1		1	1
2-3	1.31(0.788(2.17)	1.449(-0.62-3.32	0.8 (0.4-1.5)		1.69(1.1-2.5)	1.435(0.89-2.29)
4-5	1.17(0.674-2.04)	3.08(1.11-8.5)**	0.82(0.42-1.6)		1.49(0.97-2.3)	1.2(0.7-2.08)
> = 6	2.27(1.27-4.05)	2.8(0.42-7.4)	1.06(0.5-2.2)		2.3 (1.41-3.7)	1.992(1.11-3.77)**
Mother’s BMI						
<18.5	2.685( 1.762-4.09)	2.17(1.2-3.8)**	2.17(1.17 -3.81	1.25 (0.69-2.3)	3.05 (2 -4.64)	2.68(1.68-4.27)**
> = 18.5l	1	1	1	1	1	1
Source of drinking water						
Unprotected	2.5(1.63-3,88)	1.88(0.79-4.4)	1.074(0.48-2.4)	1.567(0.50 -1-4.9)	2.64(1.79-4)	1.92(1.1-3.36)
Protected	1	1	1	1	1	1

** Significant at *p* < 0.05

### Factors associated with wasting and underweight

On bivariate analysis , Children living in low land agro ecology were more likely to be underweight than those living in highlands (COR = 1.9, 95 % CI (1.3-2.7)); family size of greater than 12 was protective against underweight (COR = 0.2, 95 % CI (0.5-1)), poor initiation (for above 6months) of complementary feeding was associated with underweight (COR = 0.3, 95 % CI (0.12-0.8)); mothers above 35 years of age were less likely to have underweight child than those under 20 (COR = 0.58, 95 % CI (0.38-0.8). Presence of more than one under five children (COR = 1.5, 95 % CI (1.05-2)), Children born at home (COR = 1.78, 95 % CI (1.17-2.7)), twin births (COR = 3.9, 95 % CI (1.5-10)), presence of diarrhea (COR = 3, 95 % CI (2-4.6)) and fever in the last two weeks preceding the survey (COR = 1.9, 95 % CI (1.2-3)),having underweight mother (COR = 2.6, 95 % CI (1.7-4)) and use of unprotected source of drinking water (COR = 2.5, 95 % CI (1.6-3.8)) were also associated with underweight.

The multivariate analysis showed that Children in the lowland kebeles, (AOR = 3.29, 95 % CI (1.2-8.8)) and children having diarrhea (AOR = 2.48, 95 % CI (1.28-4.78)), children of mothers with BMI < 18.5( AOR = 2.17, 95 % CI (1.17-3.81)), children of mothers who did not have ANC visit during pregnancy ( AOR = 3.47, 95 % CI (1.49-7.8)) and children of birth order 4 to5 (AOR = 3.08, 95 % CI (1.11-8.5) were more likely to be underweight than their counterparts. Moreover, Male children were taller than female (AOR = 2.37, 95 % CI (1.19-4.7)). Children who were served food with family (AOR = 2.3, 95 % CI (1.14-4.9)), and children who had fever (AOR = 2.9, 95 % CI (1.16-7.2)) were more likely to be wasted than their counterparts. Households who used pit for garbage disposal were 87 % less likely to have wasted child than those who dispose garbage on open field (AOR = 0.13, 95 % CI (0.063-0.416)).

### Factors associated with wasting

On bivariate analysis, lack of Ante natal care (ANC) follow up was associated with having wasted child with COR = 3.2, 95 % CI (1.7-5.8), and family size of above 12 was associated with wasting, with COR = 14.8, 95 % CI(3.1-69). Male children were more likely to be wasted with COR = 1.6, 95 % CI (1.1-2.6). Prelactal feeding was associated with wasting ( COR = 2.2, 95 % CI (1.2-4.1)), and having fever was also associated with wasting (COR = 2.6, 95 % CI (91.4-4.60)).

In multivariate analysis, the odds of being wasted among male children was 2 times higher than that of female children (AOR = 2.37, 95 % CI (1.19-4.7)). Children who were served food with family and for whom food is not prepared separately were 2 times more likelyto be wasted than children who were served separately (AOR = 2.3, 95 % CI (1.14-4.9)). Mothers who had no ANC visit were 4 times more likely to have wasted child as compared to mothers who had ANC visit during pregnancy (AOR = 3.93, 95 % CI (1.35-9.6)).Children who had fever in the past two weeks, prior to the study, were 3 times more wasted (OR = 2.9, 95 % CI (1.16-7.2)). Finally, households who used garbage disposal pit were 78 % less likely to have wasted child than those who dispose on open field (AOR = 0.13, 95 % CI (0.063-0.416)).

## Discussions

The study shows that the prevalence of stunting, wasting and underweight were 45.8 %, 10.7 % and 21 % respectively. According to WHO’s classification, the prevalence of stunting in the study area is very high. Thus, children under five years in the study area have poor nutritional history and growth failure which will lead to high child morbidity and mortality. Moreover, underweight and wasting are also significantly high. Thus, a tailored nutrition education to mothers or caretakers should be given to improve the nutritional status of their children.

In this study area, stunting is higher than that of the study conducted in Gondar which was 24 % [4]. A study conducted only in rural kebeles of Haramaya district in 2010 reported a stunting prevalence of 42.2 % [5]. Even though the current study included urban kebeles the prevalence in the current study is higher. This might be due to difference in use of growth standard ( This study used WHO growth standard while a study done by Zewdu used National Centre for Health Statistics (NCHS) growth standard to get the prevalence of under nutrition). The WHO growth standard is known to increase the prevalence of under nutrition specially stunting as compared to the NCHS growth standard [19].

The prevalence of underweight and wasting in this study is 21 % and 10.7 %, respectively which is still high as per the WHO classification. However, it is lower than the study conducted in rural kebeles of Haramaya district which was 36.6 % and 14.1 % [5]; in west Gojjam which was 49.2 % and 14.8% [6], and it may be due to inclusion of urban and rural kebeles in the study, or it may also be due to improvement of the situation. A study conducted in Oromia region, Gimbi, which included urban kebeles reported comparable prevalence of underweight to this study which was 23.5 % [7].

The current study revealed that place of residence was strong predictor of stunting. And it is consistent with the study conducted in Zambia [8], and Mongolia [9]. Number of under five children in the household is significantly associated with long-term nutritional status of children. This is not surprising as the number of children under five years of age increases it may strain intra-household availability of resources and childcare practices. The findings of this study agreed with the study conducted in southern part of Ethiopia [10] and in Nigeria [11]. The finding of this study showed that the risk of stunting increases with age and this finding is in agreement with a study conducted in west Gojjam, Ethiopia [6], Uganda [13], India [14] and Vietnam [12].

Maternal nutrition influences fetal growth and birth weight which has an intergenerational link between maternal and child nutrition (UN ACC/SCN, Women and nutrition, 1990). In this study mothers with BMI < 18.5 were more likely to have stunted and underweight child than their counterparts. This finding is consistent with a study conducted in Vietnam in which underweight mothers (BMI < 18.5) were 2 times more likely to have underweight child compared to those with BMI > =18.5 (AOR = 1.95, 95 % CI (1.15, 3.33)) [12]. Another study conducted in India also showed that an increase in 1 unit of maternal BMI was associated with a lower relative risk (RR) for childhood under nutrition (underweight,(RR = 0.957,95 % CI(0.947–0.967)) stunting,(RR = 0.985,95 % CI(90.977–0.993)) wasting, (RR = 0.941,95 % CI (0.926–0.958))) [14]. And a study in Bangladesh showed that mothers whose BMI is <18.5 were 1.32 and 1.6 times more likely to have stunted and underweight child respectively as compared to those with BMI > =18.5 [12, 15, 16]. A study in India showed that in addition to underweight and stunting, higher maternal BMI was associated with lower OR for wasting (OR = 0.941) [14]. But the current study lacks significant association with wasting.

In this study Households who dispose garbage in pit were 78 % less likely to have wasted child as compared to those who dispose on open field. On the other hand a study conducted in Butajira Ethiopia and Brazil showed similar results with stunting [3, 17].

In this study, children who had diarrhea were 2 times more likely to be under weight than those who did not have. This might be because diarrhea causes dehydration and loss of appetite which is followed by decreased food intake and then malnutrition. Malnutrition by itself can cause diarrhea by decreasing absorption of nutrients. And the findings of this study agreed with a study conducted in rural Bangladesh [18].

In the current study mothers who did not visit ANC were 3.5 times more likely to have underweight child than those who had ANC visit, and a study from southern Ethiopia indicated that the number of ANC visit is linked to stunting [10]. Another study conducted in Gimbi, Ethiopia, showed that, mothers who did not attend ANC visit were more likely to have underweight child than those who had attended [7].

## Conclusions

A large proportion of under five children were stunted underweight and wasted in the study area. Thus, children are at a higher risk of under nutrition related morbidity and mortality. This study also revealed household, maternal, socio-economic, and environmental related predictors of under nutrition. Further progress in under nutrition prevention can be achieved by specifically targeting children at their early ages and conducting tailored public education to improve nutritional status of the study subjects.

### Limitation and strength of the study

#### Limitation of the study

Recall bias, under or over reporting of age of the mother and children.

#### Strength of the study

Since the study was community based and interview was conducted by going house to house, it can represent the community.

## Abbreviations

ANC: Antenatal care
AOR: Adjusted odd ratio
BMI: Body Mass Index
H/A: Height for age
HAZ: Height for age Z score
NCHS: National Centre for Health Statistics
OR: Odds ratio
SCN: Standing Committee on Nutrition
SD: Standard deviation
SPSS: Statistical package for social science
UNICEF: United National International Children's Fund
W/A: Weight for age
WAZ: Weight for age Z score
WHO: World Health Organization
